# Nonfatal and fatal cardiovascular disease events in CPAP compliant obstructive sleep apnea patients

**DOI:** 10.1007/s11325-019-01808-4

**Published:** 2019-03-08

**Authors:** Minna Myllylä, Anna Hammais, Mikhail Stepanov, Ulla Anttalainen, Tarja Saaresranta, Tarja Laitinen

**Affiliations:** 1 Division of Medicine, Department of Pulmonary Diseases, Turku University Hospital and University of Turku, Hämeentie 11, FI-20520 Turku, Finland; 2 Centre for Clinical Informatics, Hospital District of Southwest Finland, Turku, Finland; 3 Sleep Research Centre, Department of Pulmonary Diseases and Clinical Allergology, University of Turku, Turku, Finland

**Keywords:** Obstructive sleep apnea, Continuous positive airway pressure, Cardiovascular disease, Mortality

## Abstract

**Purpose:**

Obstructive sleep apnea (OSA) is suggested to predispose to cardiovascular disease (CVD) events. It is uncertain whether compliance to continuous positive airway pressure (CPAP) treatment could attenuate the risk. We explored this issue in long-term CPAP users and untreated controls.

**Methods:**

Retrospective observational cohort of CPAP-treated and control patients were pairwise matched for gender, age, and apnea–hypopnea index (AHI). The study end point was a composite of nonfatal and fatal CVD events. Cox regression model was used to determine the association between CPAP treatment and event-free survival.

**Results:**

A total of 2060 patients (75.8% male, mean age 56.0 ± 10.5 years), of which 76.4% had moderate–severe OSA, were included. In the CPAP-treated group (*N* = 1030), the median use of CPAP was 6.4 h/day during a median follow-up of 8.7 years. The control group (*N* = 1030) was followed for a median of 6.2 years after the CPAP treatment had ended. The study end point occurred in 14.4% (*N* = 148) of the CPAP-treated and in 18.8% (*N* = 194) of the control patients (*p* = 0.006). Using the Cox regression model adjusted for gender, age, AHI, body mass index, and history of CVD, hypertension, type 2 diabetes, and chronic obstructive pulmonary disease at baseline, a beneficial association between CPAP treatment and CVD risk was observed (hazard ratio 0.64, confidence interval 95% 0.5–0.8, *p* < 0.001).

**Conclusions:**

CPAP treatment was associated with a decreased risk of nonfatal and fatal CVD events. Majority of the patients were compliant to CPAP. The association was demonstrated independent from common cardiovascular risk factors and AHI.

**Electronic supplementary material:**

The online version of this article (10.1007/s11325-019-01808-4) contains supplementary material, which is available to authorized users.

## Introduction

Moderate–severe obstructive sleep apnea (OSA) has been estimated to affect 17% of middle-aged men and 9% of women [1], thus imposing a heavy burden on the health care system. OSA is characterized by recurrent episodes of complete (apneas) and partial (hypopneas) upper airway obstruction during sleep [2, 3], and it has been associated with an increased risk of cardiovascular disease (CVD) events and deaths [4].

The primary treatment for OSA is continuous positive airway pressure (CPAP), which has been shown to consolidate sleep by decreasing the number of apnea–hypopnea episodes [5]. Observational studies have demonstrated a beneficial association between CPAP treatment and CVD risk [6–9], whereas randomized controlled trials (RCT) have failed to show any significant association [10–12]. However, suboptimal adherence to CPAP has been problematic and may result in the use of the device only during the early hours of the night, thus exposing to the effects of rapid eye movement (REM) sleep which has been associated with greater number of apneas and desaturations in OSA patients [13].

The purpose of this retrospective observational study was to further evaluate the association between CPAP treatment and CVD risk in a large cohort of OSA patients, of which the majority was compliant to the treatment, compared to untreated controls.

## Methods

### Study design

We compared the occurrence of nonfatal and fatal CVD events in patients with long-term CPAP treatment (CPAP-treated patients) to patients who despite of the doctor’s advice discontinued the CPAP treatment (control patients). The groups were pairwise matched for gender, age, and AHI.

### Subjects

**Fig. 1:**
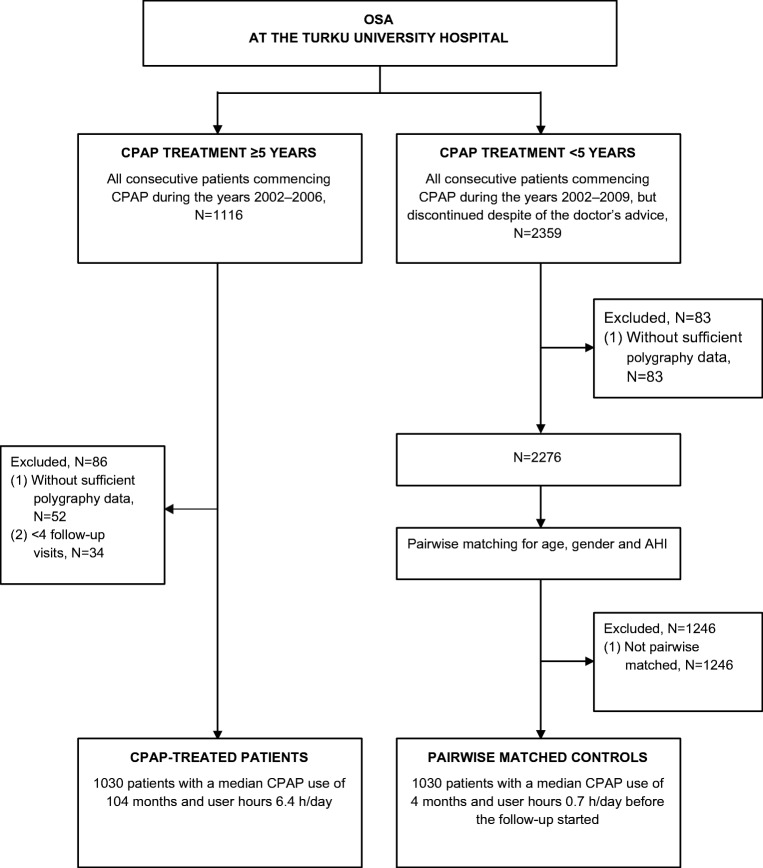
Flowchart of the recruitment of the CPAP-treated OSAS patients and their pairwise matched untreated controls

Using the discharge database of the Turku University Hospital (Turku, Finland), we identified 1116 patients who had commenced CPAP treatment due to OSA during the years 2002–2006 and continued the treatment at least for 5 years (Fig. 1). Patients without available cardiorespiratory polygraphy data were excluded (*N* = 52). From the eligible patients, 1030 had at least four follow-up visits during the treatment allowing the mean user hours to be computed for each patient with confidence. These 1030 patients formed the group of CPAP-treated patients without any further selection.

The control group was identified among those OSA patients who had commenced CPAP treatment during the years 2002–2009 (*N* = 2359), but despite of the doctor’s advice discontinued the treatment within 5 years. Patients without available cardiorespiratory polygraphy data were excluded (*N* = 83). The remaining patients (*N* = 2276) had used CPAP for a median of 8.9 (IQR 28.6) months. Among these patients, K-means clustering algorithm with Manhattan distance (absolute value distance) was used to find the best-matched control for each CPAP-treated patient in regard to gender, age and AHI. The average difference between a CPAP-treated patient and a matching control patient was 2.8 years in age and 3.5/h in AHI reported as absolute values. The pairwise matched control patients (*N* = 1030) had used CPAP for a median of 4.0 (IQR 16.0) months prior to the commencement of the study.

### Sleep studies

All included patients underwent cardiorespiratory polygraphy prior to initiation of CPAP treatment. OSA was diagnosed either by a cardiorespiratory polygraphy in the hospital (Embla, Somnologica Software, Flaga hf. Medical Devices, Reykjavik, Iceland) or by an ambulatory device at home (Embletta, Somnologica Software, Flaga hf. Medical Devices, Reykjavik, Iceland). A pulmonologist or a clinical neurophysiologist scored the sleep studies. Categories of OSA severity were defined according to the AHI: AHI < 5/h (normal), AHI ≥ 5, and < 15/h (mild OSA), AHI ≥ 15 and < 30/h (moderate OSA), and ≥ 30/h (severe OSA) [14]. Generally, CPAP treatment was recommended for patients with AHI ≥ 15/h. In patients with AHI < 15/h, assessment of the need for treatment was made individually according to patients’ symptoms and clinical findings strongly suggestive for OSA.

The scores of the self-administered Epworth Sleepiness Scale (ESS) [15] and General Health Questionnaire (GHQ-12) [16] were determined at the commencement of CPAP treatment and entered in the electronic medical records.

### Baseline comorbidity and CVD risk factors

The following baseline variables were examined from the electronic medical records: age, gender, body mass index (BMI), serum cholesterol concentration, and smoking status (defined as current smoker, ex-smoker or never smoker). Hypertension was defined as blood pressure (BP) greater than 140/90 mmHg and/or use of antihypertensive medication. CVD included coronary, cerebral, or peripheral artery diseases. An internist diagnosed coronary artery disease (CAD) by clinical symptoms and findings and/or exercise testing or coronary angiography. A neurologist confirmed the diagnosis of stroke by computerized tomography (CT) or magnetic resonance imaging (MRI). Peripheral artery disease was assessed by ankle-brachial index. Diagnosis of type 2 diabetes (T2D; fasting glucose of at least 7.0 mmol/l, use of insulin and/or oral antidiabetics) or impaired fasting glucose (IFG; fasting glucose of 6.1–6.9 mmol/l) was determined. Information of chronic obstructive pulmonary disease (COPD) diagnosed by a pulmonologist was derived from the electronic hospital records.

### Study end point and follow-up

The study end point was a composite of nonfatal (primary or secondary) and fatal CVD events. In the Cox regression model, the time to the first event was analyzed. Nonfatal CVD event was a composite of angina pectoris (stable or unstable), CAD diagnosed by angiography, and invasively treated, nonfatal myocardial infarction and nonfatal stroke. CVD events were diagnosed in special health care by cardiologists or internists according to the international guidelines. The Turku University Hospital is the only hospital in the region performing surgical and interventional cardiology procedures and having emergency department and intensive cardiac care unit. Basic cause of death included all ICD10 I-category diagnoses (I00–I99) confirmed by autopsy or documented disease history. Cause of death was derived from the national registry maintained by Statistics Finland.

Among CPAP-treated patients, the follow-up time started from the commencement of CPAP treatment and ended to the first nonfatal or fatal CVD event, all-cause death, CPAP withdrawal, or the last CPAP follow-up visit before the end of the year 2014. The median follow-up time was 8.7 (IQR 2.8) years. The control patients were followed from the last CPAP follow-up visit until the first nonfatal or fatal CVD event, all-cause death, or until the end of the year 2014. The median follow-up time was 6.2 (IQR 4.1) years.

CPAP follow-up visits occurred approximately three times during the first year and after habituation every second year. Mean number of visits during the follow-up period was 8.4 ± 2.2 per CPAP-treated patient. Treatment pressure (cmH_2_O) and CPAP usage hours (h/day) were recorded by inbuilt counter clock of the CPAP device. The use of CPAP was determined from the device at every visit undertaken during the study and entered in the electronic medical records. For each patient, the mean usage hours across the CPAP treatment period were determined. CPAP-treated patients with a median CPAP usage of at least 4 h/day were considered compliant to the treatment. Patients engaging in bariatric surgery and controls commencing mandibular advancement device (MAD) treatment during the study follow-up were identified.

### Data analysis

Data analyses were performed using the IBM SPSS Statistics 22.0 (Armonk, NY, USA: IBM Corp.) software package. Continuous variables with normal distribution were presented as mean values and standard deviations (SD), whereas variables not normally distributed were reported as median values and interquartile ranges (IQR). Means and medians were compared by using the independent-samples T test and the Mann–Whitney *U* test, respectively. Categorical variables were expressed as frequencies and percentages, and the comparisons were performed with the *χ*^2^ test. *P* values of < 0.05 were considered significant.

Cox regression model was used to evaluate the association between CPAP treatment and the occurrence of nonfatal and fatal CVD events. The adjusted model was adjusted for the following confounding covariates: gender, age, AHI, BMI, IFG/T2D, COPD, hypertension, and CVD at baseline. In the unadjusted model, each covariate was computed separately in the model. Hazard ratios (HR) and 95% confidence intervals (CI) were used to determine the associations between the studied covariates and the time to the study end point. Nonadjusted Kaplan–Meier curves with data censored at the time of the end of follow-up were used to visually compare the risk of nonfatal or fatal CVD event across the groups.

## Results

### Patients characteristics at baseline

Each CPAP-treated and corresponding control patient was pairwise matched for age, gender, and AHI at baseline (Table 1). Severe OSA was diagnosed in 498 (48.3%) of the CPAP-treated and in 454 (44.1%) of the control patients (*p* = 0.052). Moderate–severe OSA was diagnosed in 76.2% of the CPAP-treated and 76.5% of the control patients, whereas 23.8% and 23.5% of the patients had an AHI less than 15/h, respectively (*p* = 0.876). Majority of the CPAP-treated and control patients were male and obese (BMI > 30 kg/m^2^, 67.1% vs. 60.3%, *p* = 0.001, respectively). Excessive daytime sleepiness (ESS ≥ 10) was reported in 44.5% of the CPAP-treated and 37.2% of the control patients (*p* = 0.001).

**Table 1:** Characteristics of OSA patients, and comparison of variables between CPAP-treated and control patients

	CPAP-treated patients	Control patients	*P* value
	(*N* = 1030)	(*N* = 1030)	
Baseline characteristic:			
Male gender (*N*, %)	781 (75.8)	781 (75.8)	1.000
Age, years (mean, SD)	55.6 ± 9.8	56.4 ± 11.1	0.100
AHI, events/h (median, IQR)	28.0 (33.0)	27.0 (28.0)	0.108
BMI, kg/m^2^ (median, IQR)	32.7 (8.1)	31.5 (7.9)	*< 0.001*
IFG/T2D (*N*, %)	416 (40.4)	363 (35.2)	*0.016*
Hypertension^*^ (*N*, %)	788 (76.5)	724 (70.3)	*0.001*
Cholesterol^†^, mmol/l (mean, SD)	5.1 ± 1.0	5.0 ± 1.1	*0.009*
LDL, mmol/l (median, IQR)	2.9 (1.1)	2.8 (1.2)	*0.004*
Cardiovascular disease (*N*, %)	56 (5.4)	130 (12.6)	*< 0.001*^*#*^
Coronary artery disease	34	69	
Myocardial infarction	6	18	
Angina pectoris	3	5	
Stroke	10	25	
Intracranial atherosclerosis	1	0	
Peripheral artery disease	2	13	
Smoking (*N*, %)			
Current smoker	227 (22.0)	296 (28.7)	*< 0.001*^*#*^
Ex-smoker	368 (35.7)	355 (34.5)	
COPD (*N*, %)	47 (4.6)	78 (7.6)	*0.004*
ESS (mean, SD)	9.4 ± 4.7	8.3 ± 4.7	*< 0.001*
GHQ-12 (median, IQR)	2.0 (5.0)	2.0 (6.0)	0.126
CPAP treatment:			
Treatment duration^‡^, months (median, IQR)	104.0 (33.0)	4.0 (16.0)	*< 0.001*
CPAP usage^§^, h/day (median, IQR)	6.4 (2.3)	0.7 (2.6)	*< 0.001*
CPAP pressure^||^, cmH2O (mean, SD)	11.0 ± 2.2	10.5 ± 2.4	*0.011*

*OSA* obstructive sleep apnea, *CPAP* continuous positive airway pressure, *SD* standard deviation, *IQR* interquartile range, *AHI* apnea–hypopnea index, *BMI* body mass index, *IFG* impaired fasting glucose, *T2D* type 2 diabetes, *LDL* serum low-density lipoprotein (data available for 773 CPAP-treated and 703 control patients), *COPD* chronic obstructive pulmonary disease, *ESS* Epworth Sleepiness Scale (data available for 981 CPAP-treated and 986 control patients), *GHQ-12* General Health Questionnaire (data available for 912 CPAP-treated and 947 control patients)

Significant values are shown in italics

*Blood pressure greater than 140/90 mmHg and/or use of antihypertensive medication

^†^Total cholesterol level in serum (data available for 845 CPAP-treated and 738 control patients)

^‡^Time from the commencement of CPAP treatment to the last CPAP follow-up visit

^§^Median usage hours across the CPAP treatment period

^||^Mean CPAP pressure across the CPAP treatment period

^#^*P* value for the trend

Based on CVD risk factors the two groups differed from each other. The strongest risk factor, previous CVD, was diagnosed in 5.4% (*N* = 56) of the CPAP-treated and 12.6% (*N* = 130) of the control patients at baseline (*p* < 0.001). CAD was the most common CVD at baseline (3.3% in CPAP-treated and 6.7% in control patients, *p* < 0.001).

### Treatment of OSA

Among majority of the CPAP-treated patients, the compliance to CPAP was extremely good, median 6.4 (IQR 2.3) h/day across the follow-up period of 8.7 (IQR 2.8) years (Table 1). Only in 11.4% of the CPAP-treated patients, the median use was less than 4 h/day (median use 2.9 h/day, IQR 1.3). All patients in the control group were advised to commence CPAP treatment at the same clinic with same principles, but the majority of them stopped early on the treatment (median 4.0 months, IQR 16.0) and the compliance was poor (median use 0.7 h/day, IQR 2.6).

Of the control patients, 65 (6.3%) had started MAD treatment after stopping CPAP treatment. Of these patients, a CVD event was encountered in one patient during MAD treatment. The median lag time between the end of CPAP and the commencement of MAD treatment was 11.0 months (IQR 22.5). During the study follow-up, 12 (1.2%) of the CPAP-treated and 9 (0.9%) of the control patients had engaged in bariatric surgery (*p* = 0.511).

### The association between CPAP treatment and CVD risk

The study end point occurred in total of 14.4% (*N* = 148) of the CPAP-treated and in 18.8% (*N* = 194) of the control patients (*p* = 0.006) (Table 2). Similar trend was shown when fatal CVD events were analyzed separately (*N* = 22, 2.1% vs. *N* = 79, 7.7%, *p* < 0.001, respectively), but among nonfatal CVD events, no difference between the groups was observed (*N* = 126, 12.2% vs. *N* = 115, 11.2%, *p* = 0.451, respectively). Ischemic heart disease was the most common cause of CVD death (*N* = 52/101, 51.5%). More detailed data on CVD diagnostics and treatment are shown in online resource (Online Resource 1).

**Table 2:** Comparison of the first occurred nonfatal and fatal CVD events (study end point) between CPAP-treated and control patients

		CPAP-treated patients	Control patients	*P* value
		(*N* = 1030)	(*N* = 1030)	
Nonfatal	Nonfatal CVD events (*N* = 241)	126	115	0.451
	Stroke confirmed by CT/MRI	45	55	0.305
	CAD diagnosed by angiography and invasively treated	17	10	0.175
	Angina pectoris	42^†^	19^‡^	*0.003*
	Nonfatal myocardial infarction	22^§^	31^||^	0.210
Fatal	Fatal CVD events (*N* = 101)	22	79	*< 0.001*
	Ischemic heart disease	8	44	
	Stroke	4	6	
	Cardiomyopathy	3	10	
	Aortic dissection/aortic aneurysm	2	4	
	Other causes^*^	5	15	

*CPAP* continuous positive airway pressure, *CVD* cardiovascular disease, *CT* computerized tomography, *MRI* magnetic resonance imaging, *CAD* coronary artery disease

*Cardiac arrhythmias, heart valve disease, atherosclerosis, hypertension, and thrombophlebitis of deep veins

^†^Of which eight diagnosed by angiography (unstable angina pectoris)

^‡^Of which four diagnosed by angiography (unstable angina pectoris)

^§^Of which 17 diagnosed by angiography

^||^Of which 15 diagnosed by angiography

**Fig. 2:**
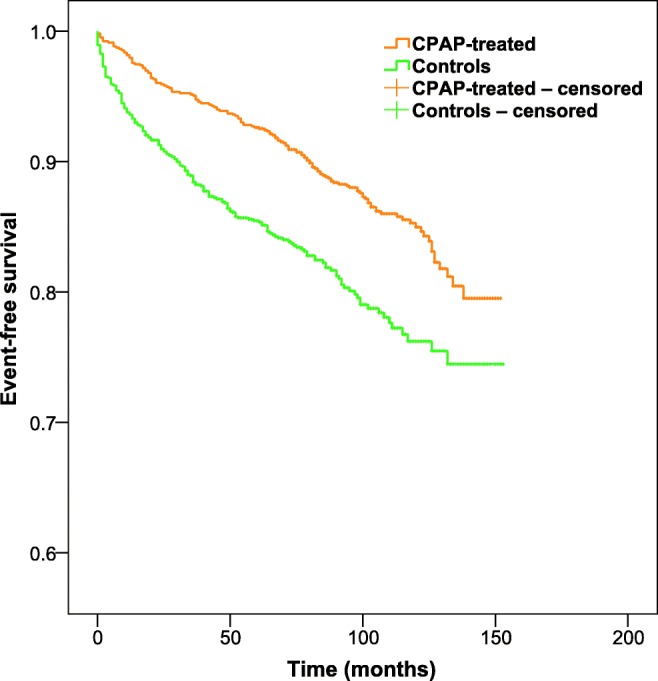
Event-free survival among CPAP-treated patients (orange, *N* = 1030) and controls (green, *N* = 1030)

CPAP treatment was associated with a decreased risk of the study end point compared to controls (HR 0.56, CI 95% 0.5–0.7, *p* < 0.001) (Table 3). The association remained significant after adjusting the model with other known CVD risk factors (HR 0.64, CI 95% 0.5–0.8, *p* < 0.001). In the adjusted model, the strongest risk factor was previous CVD (HR 4.09, CI 95% 3.1–5.3, *p* < 0.001). Also, male gender, older age, hypertension, and IFG/T2D remained independent risk factors while AHI did not. The association between CPAP treatment and reduction in CVD events was also depicted in nonadjusted Kaplan–Meier curves (Fig. 2). The association became even more significant when all CVD deaths (*N* = 33, 3.2% in CPAP-treated and *N* = 106, 10.3% in control patients, *p* < 0.001) were analyzed separately (HR 0.23, CI 95% 0.2–0.3, *p* < 0.001) (Online Resource 2).

**Table 3:** Hazard ratios and 95% confidence intervals predicting the time to the first nonfatal or fatal CVD event among CPAP-treated and control patients

	Unadjusted hazard ratios*			Adjusted hazard ratios^†^		
	HR	CI 95%	*P* value	HR	CI 95%	*P* value
Male gender	1.29	1.0–1.7	0.061	1.42	1.1–1.9	*0.014*
Age (years)	1.08	1.1–1.1	*< 0.001*	1.06	1.0–1.1	*< 0.001*
BMI (kg/m^2^)	1.01	1.0–1.0	0.272	1.01	1.0–1.0	0.304
AHI (events/h)	1.01	1.0–1.0	*0.024*	1.00	1.0–1.0	0.728
Cardiovascular disease^‡^	8.07	6.4–10.3	*< 0.001*	4.09	3.1–5.3	*< 0.001*
Hypertension^§^	2.65	1.9–3.6	*< 0.001*	1.58	1.1–2.2	*0.008*
IFG/T2D	1.94	1.6–2.4	*< 0.001*	1.47	1.2–1.8	*0.001*
COPD	2.59	1.9–3.6	*< 0.001*	1.36	1.0–1.9	0.071
CPAP treatment	0.56	0.5–0.7	*< 0.001*	0.64	0.5–0.8	*< 0.001*
Current smoker^||^	0.99	0.8–1.3	0.954	–	–	**–**
Cholesterol^#^ (mmol/l)	0.72	0.6–0.8	*< 0.001*	–	–	**–**
ESS score^#^						
Continuous	0.98	1.0–1.0	0.186	–	–	–
ESS score ≥ 10	0.90	0.7–1.1	0.347	–	–	**–**

*CVD* cardiovascular disease, *CPAP* continuous positive airway pressure, *HR* hazard ratio, *CI* confidence interval, *BMI* body mass index, *AHI* apnea–hypopnea index, *IFG* impaired fasting glucose, *T2D* type 2 diabetes, *COPD* chronic obstructive pulmonary disease, *ESS* Epworth Sleepiness Scale (data available for 981 CPAP-treated and 986 control patients)

Significant values are shown in italics

*Each covariate was computed separately in the model

^†^The model was adjusted for gender, age, BMI, AHI, cardiovascular disease, hypertension, IFG/T2D, COPD, and CPAP treatment

^‡^Coronary artery disease, myocardial infarction, angina pectoris, stroke, intracranial atherosclerosis, or peripheral artery disease

^§^Blood pressure greater than 140/90 mmHg and/or use of antihypertensive medication

^||^History of COPD instead of smoking status was used in the final adjusted model in order to better elucidate the amount of exposure

^#^Data on serum cholesterol concentrations (data available for 845 CPAP-treated and 738 control patients) and ESS score at baseline did not affect the HR of the CPAP-treated patients and were thus omitted from the final adjusted model

Of the CPAP-treated patients with median CPAP usage of more than 6 h/day (*N* = 598), 13.7% had a CVD event, whereas among patients with lower CPAP usage (*N* = 432), the corresponding incidence was 15.3% (*p* = 0.480). A significantly decreased risk of CVD event was not demonstrated among CPAP-treated patients with CPAP usage of more than 6 h/day compared to patients with lower CPAP usage (HR 0.82, CI 95% 0.6–1.1, *p* = 0.240).

## Discussion

In this large retrospective study of mainly moderate–severe OSA patients, CPAP treatment was associated with a decreased CVD risk compared to age, gender, and AHI-matched controls. The observed association was even stronger when all CVD deaths were analyzed separately. In both analyses, the association was independent from well-known CVD risk factors. Of the CPAP-treated patients, 89% were compliant to the treatment. These findings suggest that CPAP treatment may have beneficial CVD effects. The association was not related to the baseline ESS score or the severity of OSA in terms of AHI.

The most compelling evidence supports an independent causal link between OSA and hypertension. An independent association between OSA and increased risk of ischemic heart disease, stroke, cardiac arrhythmias, and mortality has also been reported [17]. In our study, majority of the patients had hypertension. It is believed that the beneficial effect of CPAP on CVD outcomes is due to its antihypertensive response [18, 19]. The level of adherence needed to achieve significant antihypertensive response is not unequivocal, but the minimum use of CPAP has been recommended to be 4 h/day and optimally more than 5 to 6 h/day [3].

The largest RCT in the field, the SAVE study, with a total of 2717 patients (mean follow-up time 3.7 years) found no protective effect of CPAP in secondary prevention. The mean CPAP usage was less than 4 h/day but a significantly lower risk of cerebrovascular event was observed when CPAP was used at least 4 h/day, even though these results were not adjusted for multiple testing [12]. Thus, it is uncertain, whether a beneficial association would have existed if the usage hours were at the recommended level. Two RCT meta-analyses in which the patients with moderate–severe OSA were randomized to either CPAP treatment or control group with medical therapy alone found that CPAP treatment of more than 4 h/day may reduce CVD risk [20, 21].

A previous meta-analysis of seven observational studies (in total of 5116 patients with a follow-up of 5–10 years) discovered that CVD death rate was significantly lower in CPAP-treated patients than in untreated severe OSA patients, and the risk of CVD death did not significantly differ from the control subjects without OSA [22]. Different OSA phenotypes not yet well known may also exist leading to different CVD risk profiles and variable cardiovascular responses to CPAP. A recent observational study of a US Veteran cohort with an average follow-up of 5 years found that particular OSA phenotypes may be associated with a greater CVD risk. Regular CPAP use (more than 4 h/day) was associated with a 36% decreased risk of adverse events and the attenuation varied by phenotypes. An increased risk of CVD was also shown in some OSA phenotypes despite the regular use of CPAP [23].

There are several strengths and potential limitations of this study that require to be addressed. The present retrospective study is one of the largest, the usage hours were well documented, and majority of the CPAP-treated patients were compliant to CPAP, possibly partly due to comprehensive patient guidance at the commencement of treatment and a rather long median treatment duration of 8.7 years indicating good adaption to CPAP device. CPAP-treated and control patients were individually matched according to gender, age, and AHI. OSA patients diagnosed with concomitant diseases were included to the study in order to better represent the real-world OSA patients. In addition to CPAP treatment, other possible treatment choices for OSA (MAD, bariatric surgery) were studied.

The retrospective study design is the most significant limitation of the present study. There can be underlying patient characteristics causing bias between CPAP-treated patients and controls. The differences between groups may include personality types. CVD and COPD were more common among controls, which might indicate that the overall lifestyle of the controls may have been unhealthier compared to the CPAP-treated patients. However, the observed association between CPAP treatment and decreased CVD risk was independent from well-known CVD risk factors such as older age, male gender, BMI, IFG/T2D, total plasma cholesterol levels, smoking, COPD, hypertension, and previous cardiovascular disease. Due to the retrospective nature of the study, data on alcohol consumption and family history of CVD were not available. The Cox regression model was adjusted for hypertension owing to the uncertainty of individual BP values.

The control group was formed in the real life setting as carefully as possible in an observational study. In order to find the best matching control for each CPAP-treated patient, the search for controls was extended by a couple of years. During these years, the treatment protocol did not fundamentally change. The shorter follow-up time of the controls should not weaken the shown association between CPAP treatment and decreased CVD risk.

A significantly decreased CVD risk could not be demonstrated among CPAP-treated patients with CPAP usage of more than 6 h/day compared to patients with lower usage. However, the overall adherence to treatment among CPAP-treated patients was extremely good leading to a relatively low dispersion in usage hours, which may have precluded the accurate estimation of the impact of CPAP adherence on CVD events. The number of occurred CVD events among CPAP-treated patients may also have been inadequate to detect a possible significant difference.

An important finding of our study is that CVD deaths were more common in the control than in the CPAP-treated group, but apart from angina pectoris, no significant differences were found in the occurrence of nonfatal CVD events. The results are consistent with a long-term follow-up study by Doherty et al. [24]. CPAP treatment may improve early signs of atherosclerosis and thus prevent the development of clinically more severe form of CVD [25].

In conclusion, our study offers further evidence that CPAP treatment might be associated with a decreased risk of nonfatal and fatal CVD events. Majority of the patients had moderate–severe OSA and were compliant to CPAP treatment. Using retrospective study design, the association was demonstrated to be independent from common cardiovascular risk factors and AHI. However, the beneficial effects of CPAP on CVD outcome remain to be confirmed in RCTs with patients compliant to the CPAP treatment.

## Electronic supplementary material

Online Resource 1 Coronary angiography data on coronary artery disease events (a component of the study endpoint) during follow-up in CPAP-treated (*N*=42) and control patients (*N*=29; *p*=0.1). (DOCX 60 kb)

Online Resource 2 Hazard ratios and 95 % confidence intervals predicting the time to cardiovascular disease death (*N*=139) among CPAP-treated and control patients. (DOCX 80 kb)

## References

[1] Peppard PE, Young T, Barnet JH, Palta M, Hagen EW, Hla KM (2013). Increased prevalence of sleep-disordered breathing in adults. Am J Epidemiol.

[2] Baguet JP, Barone-Rochette G, Tamisier R, Levy P, Pépin JL (2012). Mechanisms of cardiac dysfunction in obstructive sleep apnea. Nat Rev Cardiol.

[3] Javaheri S, Barbe F, Campos-Rodriguez F, Dempsey JA, Khayat R, Javaheri S, Malhotra A, Martinez-Garcia MA, Mehra R, Pack AI, Polotsky VY, Redline S, Somers VK (2017). Sleep apnea: types, mechanisms, and clinical cardiovascular consequences. J Am Coll Cardiol.

[4] Somers VK, White DP, Amin R, Abraham WT, Costa F, Culebras A, Daniels S, Floras JS, Hunt CE, Olson LJ, Pickering TG, Russell R, Woo M, Young T, American Heart Association Council for High Blood Pressure Research Professional Education Committee, Council on Clinical Cardiology, American Heart Association Stroke Council, American Heart Association Council on Cardiovascular Nursing, American College of Cardiology Foundation (2008). Sleep apnea and cardiovascular disease: an American Heart Association/American College of Cardiology Foundation scientific statement from the American Heart Association Council for high blood pressure research professional education committee, council on clinical cardiology, stroke council, and council on cardiovascular nursing. In collaboration with the National Heart, Lung, and Blood Institute National Center on Sleep Disorders Research (National Institutes of Health). Circulation.

[5] Leech JA, Onal E, Lopata M (1992). Nasal CPAP continues to improve sleep-disordered breathing and daytime oxygenation over long-term follow-up of occlusive sleep apnea syndrome. Chest.

[6] Marin JM, Carrizo SJ, Vicente E, Agusti AG (2005). Long term cardiovascular outcomes in men with obstructive sleep apnoea-hypopnoea with or without treatment with continuous positive airway pressure: an observational study. Lancet.

[7] Campos-Rodriguez F, Martinez-Garcia MA, Reyes-Nuñez N, Caballero-Martinez I, Catalan-Serra P, Almeida-Gonzalez CV (2014). Role of sleep apnea and continuous positive airway pressure therapy in the incidence of stroke or coronary heart disease in women. Am J Respir Crit Care Med.

[8] Martínez-García MA, Campos-Rodríguez F, Catalán-Serra P, Soler-Cataluña JJ, Almeida-Gonzalez C, de la Cruz Morón I, Durán-Cantolla J, Montserrat JM (2012). Cardiovascular mortality in obstructive sleep apnea in the elderly: role of long-term continuous positive airway pressure treatment: a prospective observational study. Am J Respir Crit Care Med.

[9] Milleron O, Pillière R, Foucher A, de Roquefeuil F, Aegerter P, Jondeau G, Raffestin BG, Dubourg O (2004). Benefits of obstructive sleep apnoea treatment in coronary artery disease: a long-term follow-up study. Eur Heart J.

[10] Barbé F, Durán-Cantolla J, Sánchez-de-la-Torre M, Martínez-Alonso M, Carmona C, Barceló A, Chiner E, Masa JF, Gonzalez M, Marín JM, Garcia-Rio F, Diaz de Atauri J, Terán J, Mayos M, de la Peña M, Monasterio C, del Campo F, Montserrat JM, Spanish Sleep And Breathing Network (2012). Effect of continuous positive airway pressure on the incidence of hypertension and cardiovascular events in nonsleepy patients with obstructive sleep apnea: a randomized controlled trial. JAMA.

[11] Peker Y, Glantz H, Eulenburg C, Wegscheider K, Herlitz J, Thunström E (2016). Effect of positive airway pressure on cardiovascular outcomes in coronary artery disease patients with nonsleepy obstructive sleep apnea. The RICCADSA randomized controlled trial. Am J Respir Crit Care Med.

[12] McEvoy RD, Antic NA, Heeley E, Luo Y, Ou Q, Zhang X, Mediano O, Chen R, Drager LF, Liu Z, Chen G, du B, McArdle N, Mukherjee S, Tripathi M, Billot L, Li Q, Lorenzi-Filho G, Barbe F, Redline S, Wang J, Arima H, Neal B, White DP, Grunstein RR, Zhong N, Anderson CS, SAVE Investigators and Coordinators (2016). CPAP for prevention of cardiovascular events in obstructive sleep apnea. N Engl J Med.

[13] Findley LJ, Wilhoit SC, Suratt PM (1985). Apnea duration and hypoxemia during REM sleep in patients with obstructive sleep apnea. Chest..

[14] (1999) Sleep-related breathing disorders in adults: recommendations for syndrome definition and measurement techniques in clinical research. The Report of an American Academy of Sleep Medicine Task Force. Sleep;22:667–89 10450601

[15] Johns MW (1991). A new method for measuring subjective daytime sleepiness: the Epworth Sleepiness Scale. Sleep.

[16] Goldberg DP, Gater R, Sartorius N (1997). The validity of two versions of the GHQ in the WHO study of mental illness in general health care. Psychol Med.

[17] Lurie A (2011). Cardiovascular disorders associated with obstructive sleep apnea. Adv Cardiol.

[18] Bazzano LA, Khan Z, Reynolds K, He J (2007). Effect of nocturnal nasal continuous positive airway pressure on blood pressure in obstructive sleep apnea. Hypertension.

[19] Martínez-García MA, Capote F, Campos-Rodríguez F, Lloberes P, Díaz de Atauri MJ, Somoza M, Masa JF, González M, Sacristán L, Barbé F, Durán-Cantolla J, Aizpuru F, Mañas E, Barreiro B, Mosteiro M, Cebrián JJ, de la Peña M, García-Río F, Maimó A, Zapater J, Hernández C, Grau SanMarti N, Montserrat JM, Spanish Sleep Network (2013). Effect of CPAP on blood pressure in patients with obstructive sleep apnea and resistant hypertension: the HIPARCO randomized clinical trial. JAMA.

[20] Abuzaid AS, Al Ashry HS, Elbadawi A (2017). Meta-analysis of cardiovascular outcomes with continuous positive airway pressure therapy in patients with obstructive sleep apnea. Am J Cardiol.

[21] Khan SU, Duran CA, Rahman H, Lekkala M, Saleem M, Kaluski E (2017). A meta-analysis of continuous positive airway pressure therapy in prevention of cardiovascular events in patients with obstructive sleep apnoea. Eur Heart J.

[22] Fu Y, Xia Y, Yi H, Xu H, Guan J, Yin S (2017). Meta-analysis of all-cause and cardiovascular mortality in obstructive sleep apnea with or without continuous positive airway pressure treatment. Sleep Breath.

[23] Zinchuk AV, Jeon S, Koo BB, Yan X, Bravata DM, Qin L, Selim BJ, Strohl KP, Redeker NS, Concato J, Yaggi HK (2018). Polysomnographic phenotypes and their cardiovascular implications in obstructive sleep apnoea. Thorax.

[24] Doherty LS, Kiely JL, Swan V, McNicholas WT (2005). Long-term effects of nasal continuous positive airway pressure therapy on cardiovascular outcomes in sleep apnea syndrome. Chest.

[25] Drager LF, Bortolotto LA, Figueiredo AC, Krieger EM, Lorenzi GF (2007). Effects of continuous positive airway pressure on early signs of atherosclerosis in obstructive sleep apnea. Am J Respir Crit Care Med.

