# Supplementary material for: Nonfatal and fatal cardiovascular disease events in CPAP compliant obstructive sleep apnea patients
Source: Sleep Breath. 2019 Mar 8;23(4):1209–17. doi: 10.1007/s11325-019-01808-4 (PMC6868046; doi:10.1007/s11325-019-01808-4)
Supplement: Supplementary file 1 — Coronary angiography data on coronary artery disease events (a component of the study endpoint) during follow-up in CPAP-treated (N=42) and control patients (N=29; p=0.1). (DOCX 60 kb) [file 11325_2019_1808_MOESM1_ESM.docx]

|  | **CPAP-treated**  **patients**  **(N=42/1030)** | **Control**  **patients**  **(N=29/1030)** |
| --- | --- | --- |
| Findings in angiography  CAD diagnosed and invasively treated  Unstable angina pectoris  Nonfatal myocardial infarction | 17  8  17 | 10  4  15 |
| Number of occluded coronary arteries  Three arteries or the left main  Two arteries  One artery | 11  13  18 | 7  8  14 |
| Treatment  Percutaneous coronary intervention  Coronary artery bypass grafting  Heart valve procedure  Conservative treatment | 26  11  3  2 | 22  4  0  3 |

CPAP, continuous positive airway pressure; CAD, coronary artery disease.
