# Supplementary material for: Nonfatal and fatal cardiovascular disease events in CPAP compliant obstructive sleep apnea patients
Source: Sleep Breath. 2019 Mar 8;23(4):1209–17. doi: 10.1007/s11325-019-01808-4 (PMC6868046; doi:10.1007/s11325-019-01808-4)
Supplement: Supplementary file 2 — Hazard ratios and 95 % confidence intervals predicting the time to cardiovascular disease death (N=139) among CPAP-treated and control patients. (DOCX 80 kb) [file 11325_2019_1808_MOESM2_ESM.docx]

|  | **Adjusted hazard ratios^*^** | | |
| --- | --- | --- | --- |
|  | **HR** | **CI 95 %** | **P value** |
| Male gender | 1.61 | 1.0­–2.5 | **0.039** |
| Age, years | 1.08 | 1.1–1.1 | **<0.001** |
| BMI, kg/m² | 1.02 | 1.0–1.1 | 0.158 |
| AHI, events/h | 1.01 | 1.0–1.0 | **0.001** |
| Cardiovascular disease^†^ | 3.33 | 2.2–4.9 | **<0.001** |
| Hypertension^‡^ | 1.69 | 0.9–3.0 | 0.075 |
| IFG/ T2D | 1.42 | 1.0–2.0 | 0.052 |
| COPD | 1.16 | 0.7–1.9 | 0.559 |
| CPAP treatment | 0.23 | 0.2–0.3 | **<0.001** |

HR, hazard ratio; CI, confidence interval; CPAP, continuous positive airway pressure; AHI: apnea-hypopnea index. BMI: body mass index. IFG: impaired fasting glucose. T2D: type 2 diabetes. COPD: chronic obstructive pulmonary disease.

* The model was adjusted for gender, age, BMI, AHI, cardiovascular disease, hypertension, IFG/ T2D, COPD and CPAP treatment.

† Coronary artery disease, myocardial infarction, angina pectoris, stroke, intracranial atherosclerosis or peripheral artery disease.

‡ Blood pressure greater than 140/90 mmHg and/ or use of antihypertensive medication.
